# Evaluation of the Impact of Hydrogen Peroxide on ANFO-Based Materials’ Morphology

**DOI:** 10.3390/ma18184254

**Published:** 2025-09-11

**Authors:** Andrzej Biessikirski, Michał Dworzak, Magdalena Ziąbka, Krzysztof Polak, Mateusz Pytlik, Bogna Daria Napruszewska, Łukasz Kuterasiński

**Affiliations:** 1 Faculty of Civil Engineering and Resource Management, AGH University of Krakow, al. A. Mickiewicza 30, 30-059 Krakow, Poland; dworzak@agh.edu.pl (M.D.); kpolak@agh.edu.pl (K.P.); 2 Department of Ceramics and Refractories, Faculty of Materials Science and Ceramics, AGH University of Krakow, 30-059 Krakow, Poland; ziabka@agh.edu.pl; 3 Conformity Assessment Body, Central Mining Institute-National Research Institute, 1 Gwarków Square, 40-166 Katowice, Poland; mpytlik@gig.eu; 4 Jerzy Haber Institute of Catalysis and Surface Chemistry, Polish Academy of Sciences, ul. Niezapominajek 8, 30-239 Kraków, Poland; bogna.napruszewska@ikifp.edu.pl

**Keywords:** hydrogen peroxide, ANFO, SEM, XRD, TG/DSC, morphology, energy

## Abstract

The decomposition of high-energy materials often releases large volumes of toxic fumes, contributing to environmental pollution. To reduce these emissions, eco-friendly formulations are being developed by modifying chemical composition or adding functional additives that enhance combustion and reduce toxic byproducts. Hydrogen peroxide (H_2_O_2_), acting as both an oxidizer and potential fuel, shows promise in lowering NO_x_ emissions. However, its impact on formulation stability must be assessed. This study examines the morphological and thermal behavior of an ammonium nitrate, fuel oil, and hydrogen peroxide (ANFOHP) formulation using scanning electron microscopy (SEM), X-ray diffraction (XRD), Fourier transform infrared spectroscopy (FT-IR), and thermal analysis based on thermogravimetry (TG) connected with differential scanning calorimetry (DSC) techniques. SEM showed that the fuel oil–hydrogen peroxide (FOHP) blend formed a thin film on ammonium nitrate prills without structural damage. XRD patterns indicated an intact crystalline structure. Moreover, FT-IR analysis performed both for fresh and 24-h stored samples evidenced no structural changes. In turn, TG/DSC revealed altered thermal behavior, with a new endothermic peak near 80 °C corresponding to the simultaneous evaporation of water and hydrogen peroxide from the ANFO surface, and reduced intensity of the main ANFO decomposition peak, indicating a shift in the thermal behavior induced by the FOHP blend.

## 1. Introduction

Ammonium nitrate fuel oil (ANFO) is one of the most widely employed high-energy materials in mining operations due to its simplified formulation, ability to tailor material properties, and substantial energy release during decomposition reactions [1]. This energy release, manifested through the generation of post-blast gases, facilitates the fragmentation of large volumes of output. However, it is crucial to acknowledge the environmental and human health implications of these emissions, primarily consisting of NO_x_ and CO_x_ [2,3,4].

Oluwoye et al. estimated that global annual NO_x_ emissions from ammonium nitrate (AN)-based high-energy materials amount to approximately 0.05 Tg (50,000 tons) of nitrogen, with localized NO_x_ plumes reaching concentrations of up to 500 ppm, exceeding occupational exposure limits by up to 3000 times [5]. Considering that the decomposition of 1 kg of high-energy material can generate up to 1000 dm^3^ of gaseous emissions, and given the annual consumption of 46.13 million kg of energetic materials in 2022 in the Polish mining market alone [6], Oluwoye et al.’s predictions in [5] may underestimate the true environmental impact. These findings underscore the need to develop more eco-friendly, high-energy materials, potentially through the incorporation of new additives or the elimination of undesirable components to achieve a zero-oxygen balance.

Recent research indicates promising advancements in modifying ANFO to mitigate emissions. Kuterasiński et al. evidenced that the addition of zeolite Y to ANFO reduced post-blast fume emission. Nevertheless, this action enhanced the detonation properties of such prepared ANFO materials [7]. Similarly, Biessikirski et al. explored microstructured charcoal additives, both pure and incrustated with nano-iron oxide, which lowered NO_x_ emissions without compromising the high-energy materials’ performance. The catalytic effect of iron accelerated the decomposition of ammonium nitrate, reducing harmful byproducts [8,9]. Furthermore, Lissianski et al. conducted combustion studies, revealing that metal-containing compounds can catalytically reduce NO_x_ formation [10]. Similarly, Skrelec et al. investigated fuel-rich high-energy materials through increased fuel oil content or additives such as pulverized coal (PPC) dust or aluminum powder. These modifications reduced NO_2_ emissions while sometimes slightly increasing CO levels. For instance, adding 8% excess diesel fuel or 3% PPC to ANFO significantly reduced NO_2_ emissions. Other additives forming protective gels against fuel loss have also demonstrated efficacy in maintaining reduced NO_x_ emissions [11]. Yi et al. highlighted the potential of specific inhibitors in reducing harmful gas emissions, achieving reductions of up to 50% or more depending on the type and concentration of the additives [12]. Furthermore, Barański et al., Kalombo et al., and Gerlich et al. proposed incorporating either bismuth- and antimony- or silicon-based composition into time-delay elements to create lead-free detonators, addressing occupational safety and environmental concerns [13,14,15].

Another emerging approach involves developing hydrogen-based high-energy materials by incorporating hydrogen peroxide (H_2_O_2_) [16,17,18]. Hydrogen peroxide, a highly reactive oxidizing agent, is extensively used in industrial applications and serves as a precursor for high-energy materials such as triacetone triperoxide (TATP) and hexamethylene triperoxide diamine (HMTD) [19,20,21]. Historical studies, including early 20th-century German research, examined hydrogen peroxide/fuel mixtures, revealing their detonation velocities and the influence of initiation methods. Recent evaluations by Rarata and Smętek reviewed the application of H_2_O_2_ in rocket monopropellants and oxidizers, suggesting its potential use in mining high-energy materials [17,18].

Hydrogen peroxide-based high-energy materials, including liquid or gel formulations blended with fuels such as ethanol, methanol, nitromethane [22], or nitrates like sodium, calcium, and potassium [16], have shown promise in reducing NO_x_ emissions while maintaining stable decomposition processes. However, these studies have primarily focused on liquid and gel-type high-energy materials, leaving unexplored the potential application of hydrogen peroxide as an additive in ANFO formulations.

The aim of this paper is to perform a preliminary morphological evaluation to assess whether FOHP blends could negatively impact ANFO properties. This type of research on the impact of FOHP on AN has never been conducted. The SEM, XRD, and TG/DSC analysis will allow us to check the non-ideal high-energy material structure and characteristics. Additionally, blasting properties (also called detonation properties) were determined using specialized software. Incorporating both fuel oil (FO) and hydrogen peroxide (HP) within the maximum absorption range of ammonium nitrate (AN) could potentially lead to further reductions in NO_x_ emissions, marking a significant advancement in the quest for environmentally sustainable high-energy materials.

## 2. Materials and Methods

### 2.1. Materials

Ammonium nitrate (AN) was produced by Yara International ASA, Szczecin, Poland. The AN sample was a porous prill, type 7, obtained in 2024. The material exhibited a purity of 99.5% and a bulk density of 0.81 kg∙dm^−3^. The moisture content was approximately 0.3%, and the average prill diameter measured 0.8 mm.

Diesel fuel oil (FO), produced by Orlen S.A., Płock, Poland, in 2025, was characterized by a density of 0.845 kg∙dm^−3^ and a viscosity of 3.00 mm^2^∙s^−1^.

Hydrogen peroxide (HP), supplied by Warchem Sp. z o.o., Zakręt, Poland, from a batch obtained in 2025, exhibited a relative density of 1.24 kg∙dm^−3^ and a viscosity of 1.21 mm^2^∙s^−1^.

Non-ideal high-energy materials were prepared by blending ammonium nitrate with fuel oil according to the weight ratios specified in Table 1. Before mixing, the fuel oil was combined with hydrogen peroxide and stirred for 5 min at 250 revolutions per minute (r.p.m.). The resulting fuel oil–hydrogen peroxide mixture (FOHP) was then incrementally introduced into the ammonium nitrate and thoroughly blended for 20 min at 250 r.p.m. The chemical composition of the manufactured non-ideal high-energy material samples is detailed in Table 1.

### 2.2. Methods

Scanning electron microscopy (SEM) analysis was carried out utilizing a Nova NanoSEM 200 (FEI, Eindhoven, The Netherlands). SEM images were acquired at magnifications ranging from 150× to 5000×. Observations were conducted under low-vacuum conditions (~60 Pa) employing a low vacuum detector (LVD) operating in secondary electron imaging mode. The electron beam was maintained at an accelerating voltage of 15 kV throughout the analysis. Before analysis, each sample was coated with carbon.

Structural characterization of the synthesized materials was carried out using a Nicolet iS10 Fourier transform infrared (FT-IR) spectrometer (Thermo Scientific, Waltham, MA, USA) equipped with a mercury cadmium telluride (MCT) detector operating in attenuated total reflectance (ATR) mode. Spectra were recorded over the wavenumber range of 4000–650 cm^−1^ with a spectral resolution of 4 cm^−1^. Each spectrum was obtained as an average of 128 scans. Furthermore, the impact of the addition of fuel oil on the structure of ammonium nitrate (V) was investigated. Additional evaluation of the samples ANFO, ANFO-HP-5, and ANFO-HP-10 was performed after 24 h of storage to verify potential FOHP interaction.

X-ray powder diffraction (XRD) patterns were recorded using a PANalytical X’Pert PRO MPD system (PANalytical B.V., Almelo, The Netherlands). The CuKα radiation was generated at 40 kV and 30 mA. XRD scans were conducted over a 2θ range of 5° to 50°, with a step size of 0.25°. All analyzed non-ideal high-energy material samples were positioned in sample holders. The XRD measurements were performed under ambient conditions. Moreover, the influence of the introduction of fuel oil on the crystallinity of ammonium nitrate (V) was studied.

Thermogravimetric and differential scanning calorimetry (TG-DSC) analyses were conducted within a temperature range of 20–700 °C. The experiments were carried out under an air atmosphere with a controlled flow rate of 30 mL/min, simulating detonation conditions both within the furnace and in the balance chamber. The heating rate was maintained at 10 °C·min^−1^. For each experiment, a 20 mg sample was placed into a DSC aluminum pan using a spatula immediately before analysis. Baseline corrections for TG measurements were performed using an empty pan subjected to the same heating profile. The observed TG drift was approximately 5 μg, corresponding to 0.02 wt.%.

The theoretical properties of the high-energy materials were calculated using the EXPLO5 thermochemical computational code developed by OZM Research s.r.o. (Hrochův Týnec, Czech Republic). EXPLO5 is a versatile tool for predicting energetic properties, explosibility, and performance indicators of explosives, propellants, and pyrotechnics using chemical formula, heat of formation, and density. It features a large reactant and product database. Thermodynamic calculations were based on the theory of detonation according to the Chapman–Jouguet (CJ) and Becker–Kistiakowsky–Wilson (BKW) models, which assumed instantaneous chemical reactions and thermodynamic equilibrium of the detonation products. The detonation pressure, detonation temperature, and fume volume were determined based on these thermodynamic models.

## 3. Results and Discussion

SEM imaging of ammonium nitrate (V) (AN) presents specific challenges due to its inherent hygroscopic nature and susceptibility to thermal decomposition. Exposure to the electron beam, particularly at high magnifications, may result in localized heating, leading to the decomposition of AN into nitrous oxide and water. This phenomenon can induce structural and surface alterations during imaging, compromising the accuracy and reproducibility of the observations. Furthermore, AN’s strong affinity for atmospheric moisture exacerbates surface instability, further complicating imaging procedures [23].

**Figure 1:**
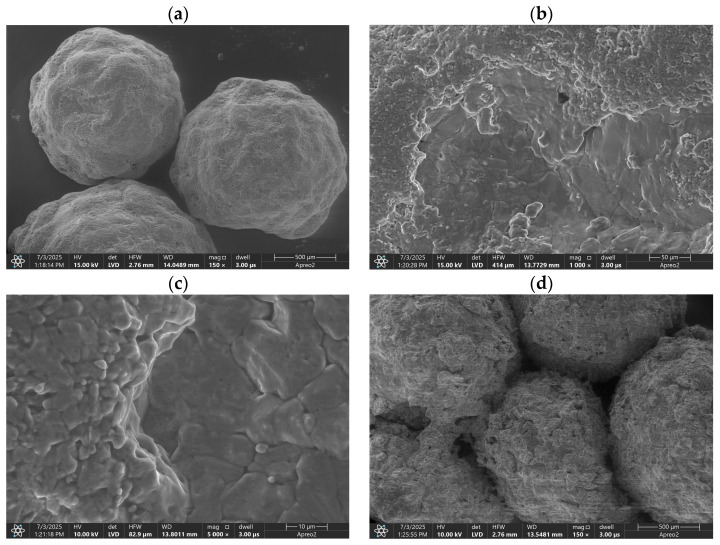

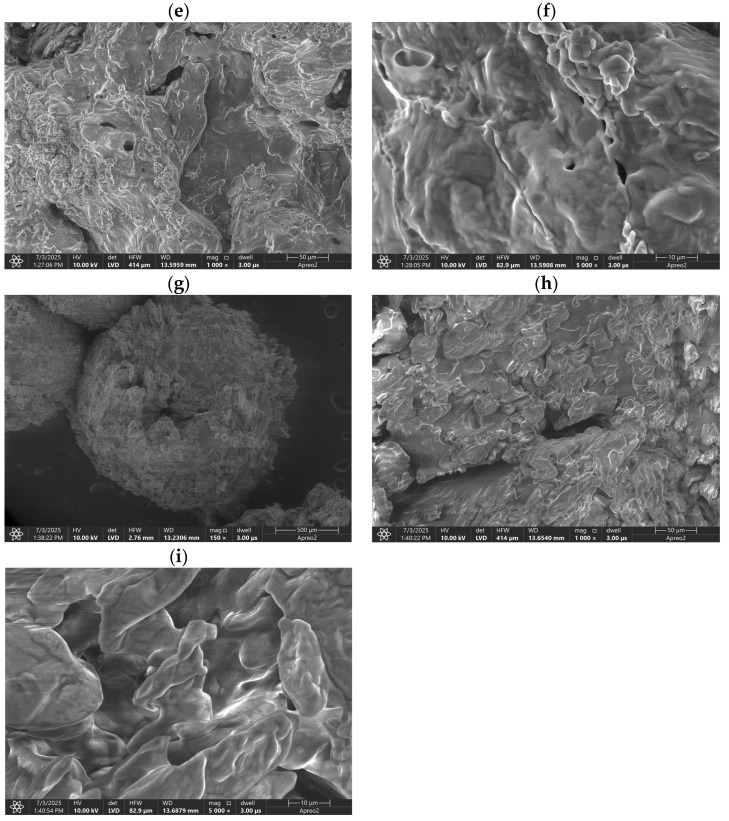
SEM images of ANFO under (**a**) 150×, (**b**) 1000×, and (**c**) 5000×; ANFO-HP-5 under (**d**) 150×, (**e**) 1000×, and (**f**) 5000×; and ANFO-HP-10 under (**g**) 150×, (**h**) 1000×, and (**i**) 5000×.

To mitigate these limitations, a carbon conductive coating, as described in Section 2, Materials and Methods, was applied to all analyzed samples. In addition, high-speed imaging protocols were employed to minimize beam-induced damage and thermal artifacts, thereby reducing the potential for crystal surface alteration and effectively excluding the influence of the HP. Representative SEM micrographs of the AN prills, wherein beam interaction did not result in visible morphological changes, are shown in Figure 1.

Previous investigations [24,25,26,27] have demonstrated that the surface morphology of AN crystals is notably irregular, exhibiting a wrinkled topology with extensive surface deformations. These deformations often manifest as intersecting and merging cracks. The provenance of the starting material can determine the occurrence and location of a central cavity [28]. Additionally, numerous surface pores are typically present. This porous, wrinkled morphology enhances the specific surface area available for contact with fuel components. Biessikirski et al. [26,27] reported open-type porosity in AN prills, which increases inert surface area in cross-sectional views and facilitates fuel absorption via steam-like microchannels, a structure corroborated by the findings of Lotspeich and Petr [24]. These structural features contribute to the high absorption capacity of AN prills.

**Figure 2:**
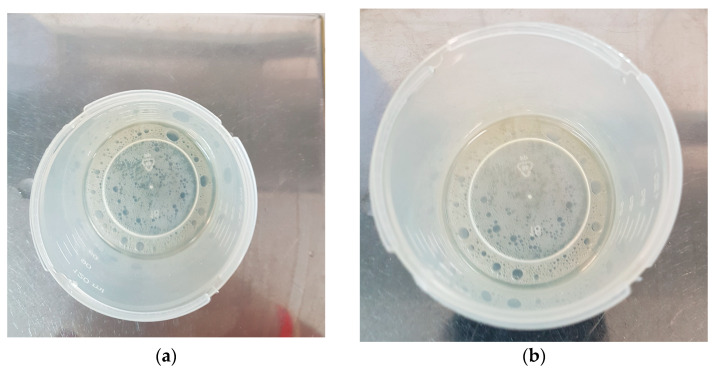
FOHP blends after 24 h of storage for (**a**) ANFO-HP-5 and (**b**) ANFO-HP-10.

Initial SEM imaging at a magnification of 150× revealed that all examined AN prills exhibited a wrinkled surface morphology with no discernible presence of either FO or the FOHP blend, as depicted in Figure 1a,d,g. Notably, some prills displayed prominent internal voids or air gaps (Figure 1g), potentially indicative of central cavity formation. At a higher magnification of 1000×, a thin surface layer attributable to either FO (Figure 1b) or FOHP (Figure 1e,h) was identified on certain prill surfaces. Both fuel types (fuel oil and hydrogen peroxide) appeared to adhere to the AN surface and penetrate surface irregularities (Figure 1f,i), likely via absorption through the aforementioned porous channels. SEM imaging at 5000× magnification revealed no appreciable morphological differences between AN surfaces treated with FO (Figure 1c) and those treated with FOHP (Figure 1f,i). These results suggest that FOHP does not induce any significant alterations in AN crystal morphology under the examined conditions. However, it is important to consider that HP, particularly in concentrated forms, may react with AN or alter the properties of FO. Reactions between AN and HP are known, with their nature and extent being contingent on parameters such as compound concentration, the presence of other substances, and temperature [29,30]. In contrast, the interaction of HP with FO may enhance the latter’s reactivity and combustion efficiency, potentially leading to more complete and energetically favorable combustion processes [31]. These chemical and physical modifications could have important implications for the formulation and performance of high-energy materials. Notably, differences in viscosity and density between HP and FO were observed. However, no issues with blend stability or phase separation were detected during a 24-h storage period, which was estimated by both optical observation (Figure 2a,b) and no changes in the appearance of the FT-IR spectra recorded for the samples containing FO-HP mixture (ANFO-HP-5 and ANFO-HP-10), either fresh or after 24-h storage (Figure A2).

Furthermore, the adherence of the FOHP mixture to the AN surface, confirmed via SEM analysis, suggests that the blend’s viscosity was sufficient to ensure homogenous coverage of the prill surface.

**Figure 3:**
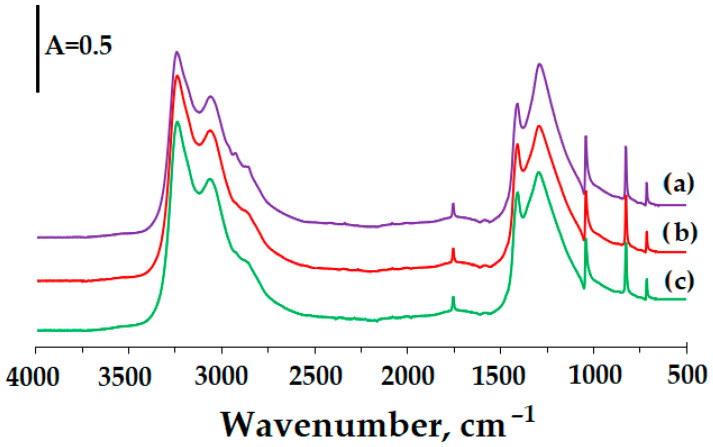
FT-IR bands for (a) ANFO, (b) ANFO-HP-5, and (c) ANFO-HP-10 samples.

The FT-IR spectra of all investigated samples (Figure 3) exhibit absorption bands in the range of 3240–2850 cm^−1^, which are assigned to the asymmetric stretching and deformation vibrations of ammonium cations [32,33]. Weak signals observed between 2950 and 2850 cm^−1^ are indicative of –CH_2_– and –CH_3_ stretching vibrations, which can be attributed to residual fuel oil [34]. The band detected at 1755 cm^−1^ may be ascribed either to the stretching vibration or to the in-plane deformation of nitrate anions; alternatively, it may arise from a combination of the asymmetric deformation mode of NH_4_^+^ with a lattice vibration. Characteristic bands at 1410 and 1290 cm^−1^ correspond to the triply degenerate deformation of NH_4_^+^ and the doubly degenerate stretching vibration of NO_3_^−^, respectively. Furthermore, the absorption maxima at 1040, 825, and 715 cm^−1^ are associated with the symmetric stretching vibration and with out-of-plane and in-plane deformation modes of nitrate anions [32,33]. Upon the addition of hydrogen peroxide, a progressive attenuation of the absorption bands corresponding to the organic phase of ANFO was observed, with this effect becoming more pronounced as the H_2_O_2_ content increased in the analyzed samples.

**Figure A1:**
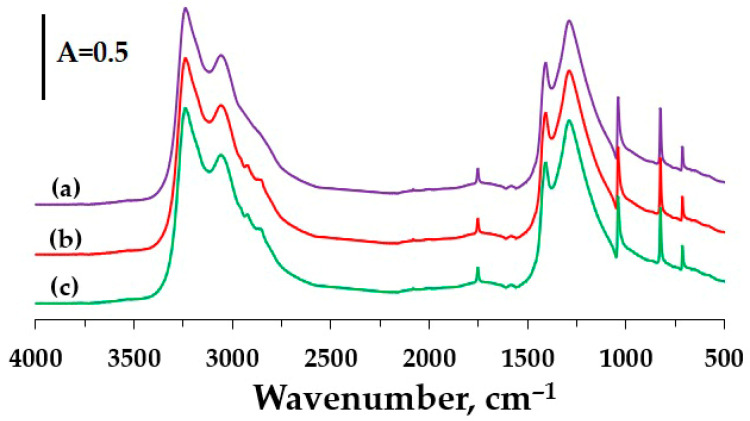
FT-IR spectra of (a) ammonium nitrate (V), (b) ANFO, and (c) ANFO after 24-h storage.

**Figure A2:**
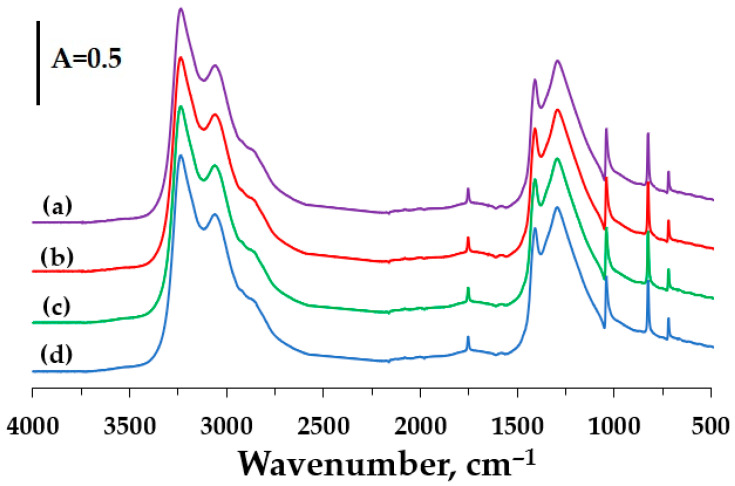
FT-IR spectra of (a) ANFO-HP-5, (b) ANFO-HP-5 after 24-h storage, (c) ANFO-HP-10, and (d) ANFO-HP-10 after 24-h storage.

The influence of the addition of fuel oil on the structure of pure ammonium nitrate (V) was investigated. The FT-IR spectra are depicted in Figure A1. From the comparison between FT-IR spectra for AN and ANFO samples, it may be concluded that the introduction of fuel oil (FO) to ammonium nitrate (AN) caused the appearance of the bands attributed to the organic phase. Furthermore, the 24-h aging procedure did not alter the structure of the ANFO sample. The same tendencies were observed for ANFO-HP-5 and ANFO-HP-10 samples, containing hydrogen peroxide (HP), which evidenced the stability of the FO-HP mixture (Figure A2).

**Figure 4:**
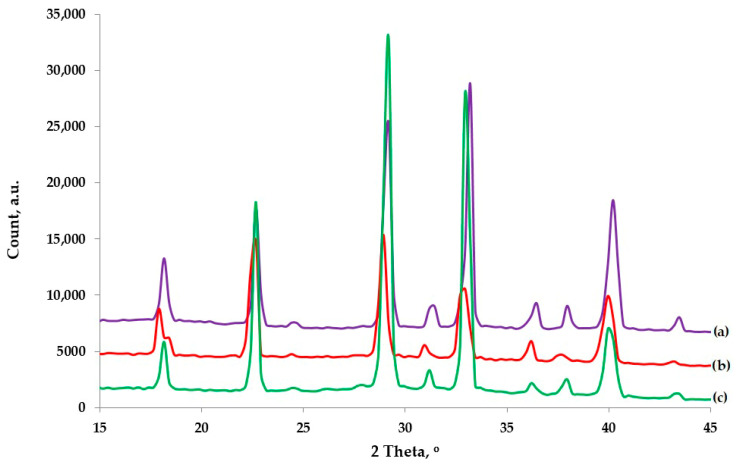
XRD patterns for (a) ANFO, (b) ANFO-HP-5, and (c) ANFO-HP-10 samples.

Although SEM imaging indicated that FOHP did not alter AN’s crystalline structure, further structural verification was conducted using XRD. The corresponding XRD patterns are presented in Figure 4, confirming the preservation of AN’s crystalline integrity following contact with FOHP.

The analysis of the XRD patterns (Figure 4) revealed a high degree of similarity across all samples, suggesting that the FOHP additive does not adversely interact with the surface of AN crystals. Existing research [35] indicates that the dominant influence in non-ideal high-energy material samples is attributed to the XRD patterns of pure AN.

The diffraction peaks observed, as illustrated in Figure 4, occur at 2θ values of 18°, 22°, 24°, 29°, 31°, 33°, 36°, and 40°, corresponding to the crystal planes (100), (011), (110), (111), (002), (020), (102), and (112), respectively [35,36,37,38]. All observed diffraction peaks correspond to ammonium nitrate (V) phase IV, the orthorhombic room-temperature polymorph that predominates in AN-based energetic formulations. When HP was introduced to ANFO (ANFO-HP-5 and ANFO-HP-10), all ammonium nitrate (V) samples exhibited identical crystal symmetry and diffraction patterns, as depicted in Figure 4. In ANFO-HP-10 (Figure 4), the XRD result exhibits sharp, high-intensity reflections, particularly in the 30–34° 2θ region, whereas ANFO displays comparatively broader, lower-intensity peaks on an elevated background. This systematic progression reflects the development of a long-range crystallographic order, characterized by larger coherent domain sizes and reduced microstrain, as the proportion of H_2_O_2_/water in the formulation increases. The FO component remains amorphous and does not generate crystalline reflections; instead, it acts primarily as a diluent or surface film on AN crystallites. Consequently, the crystalline signature is governed exclusively by AN, with only minor contributions from amorphous scattering. The gradual substitution of AN by HP introduces substantial water into the system. Water is well known to mediate AN polymorphic transformations and recrystallization, favoring the growth of well-faceted AN-IV crystals upon drying and influencing the kinetics of the IV ↔ III phase transition. The monotonic sharpening of diffraction peaks observed from ANFO to ANFO-HP-10 (Figure 4) is therefore consistent with a partial dissolution recrystallization mechanism, whereby moisture and H_2_O_2_ promote preferential growth of AN crystallites. This process enhances the preferred orientation in specific lattice planes, explaining the disproportionate intensification of the 30–34° reflections [39,40,41,42]. Importantly, the addition of FO–HP mixtures to AN (ANFO-HP-5 and ANFO-HP-10) does not produce any additional diffraction peaks relative to ANFO that would indicate the formation of a novel peroxide-containing crystalline phase distinct from AN-IV. Although peroxide-based peroxosolvates and adducts are known in certain chemical systems, their formation with simple nitrate salts under ambient processing conditions is highly improbable and generally requires specific co-formers or specialized conditions. Thus, the persistence of AN-IV as the sole crystalline phase across all samples is consistent with expectations [43,44,45].

In summary, all three formulations are dominated by ammonium nitrate (V) phase IV. Increasing the fraction of 60% H_2_O_2_ (and the associated water content) induces recrystallization, leading to enhanced apparent crystallinity, as evidenced by the sharper and more intense AN-IV reflections in ANFO-HP-10. No crystalline peroxide-containing phases were detected, and FO remained entirely amorphous. These structural changes align with the established literature on water-mediated AN polymorphism and crystallization and hold practical significance for FO uptake, storage stability, and moisture management in ANFO–H_2_O_2_ energetic formulations [46,47]. This finding indicates that blend additives such as FOHP do not modify the intrinsic crystal structure of ammonium nitrate (V). The retention of crystal symmetry and diffraction patterns further confirms that fuel oil absorption occurs within the porous framework of AN prills without inducing phase transitions or lattice distortions.

**Figure A3:**
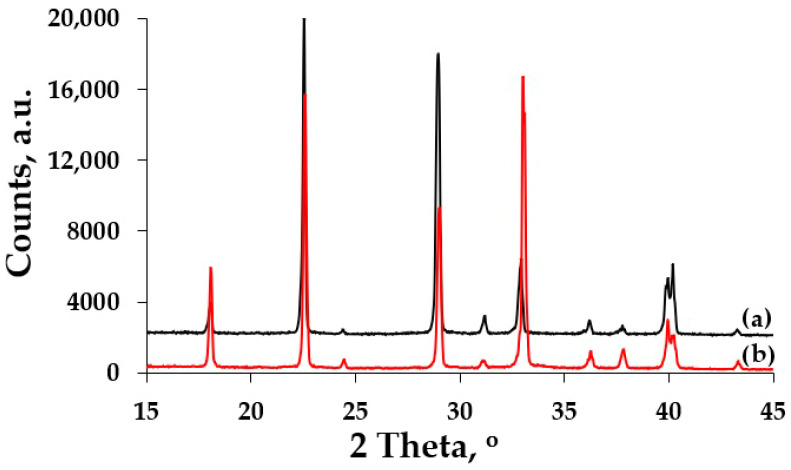
XRD patterns of (a) ammonium nitrate (V) and (b) ANFO.

The influence of the addition of fuel oil on the crystallinity of pure ammonium nitrate (V) was investigated. The XRD patterns are illustrated in Figure A3. Direct comparison between XRD patterns for AN and ANFO samples did not indicate any relevant changes in crystallinity as a result of the addition of fuel oil to ammonium nitrate (V).

**Figure 5:**
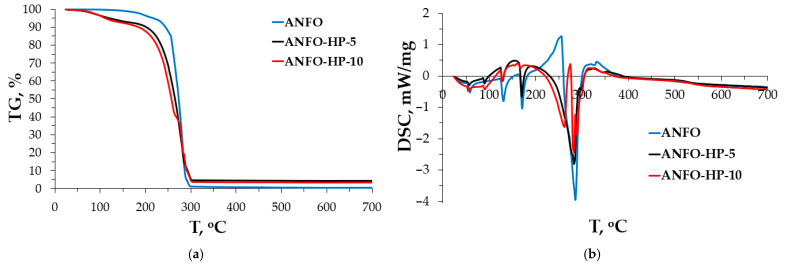
Results of (**a**) TG and (**b**) DSC analysis of tested samples.

As was mentioned, the HP addition may result in more favorable combustion processes, which may subsequently result in the modification of high-energy materials’ performance. To establish this, the TG/DSC measurements and EXPLO5 simulations were performed. The TG/DSC results are presented in Figure 5.

Figure 5 presents the TG and DSC profiles recorded in the temperature range of 20–700 °C for samples prepared by mixing AN with FO and a 60% aqueous HP solution. For the reference ANFO sample, the TG curve (Figure 5a) reveals an initial mass loss below 200 °C, attributed to the evaporation of fuel oil from the AN matrix [47]. Upon further heating, the sample underwent complete mass loss, corresponding to the thermal decomposition of AN, as described by the following reaction (1) [48]:

NH_4_NO_3_ → N_2_O + 2H_2_O (1)

The TG profile’s shape (Figure 5a) was dependent on the chemical composition of the respective ANFO formulation. In the case of samples with reduced fuel oil content and the presence of aqueous hydrogen peroxide (samples ANFO-HP-5 and ANFO-HP-10), a more pronounced mass loss was observed below 200 °C compared to the ANFO sample. This enhanced weight loss is likely due to the relatively facile evaporation of the low-boiling, inorganic components, namely water and hydrogen peroxide, compared to the more complex, organic constituents of fuel oil [49,50,51]. Moreover, TG curves for H_2_O_2_-containing samples—particularly ANFO-HP-10—exhibited inflection points in the 250–280 °C and ~290 °C temperature ranges. These features are suggestive of chemical decomposition processes involving hydrogen peroxide, most notably its conversion into water and oxygen. Notably, the decomposition onset occurred at lower temperatures than pure hydrogen peroxide (typically ~450 °C), which is attributable to the aqueous environment in which it was dissolved [52]. Complete decomposition was not achieved in these H_2_O_2_-containing samples, with residual masses of approximately 5% of the original sample weight remaining at the end of the analysis.

The DSC curves reveal distinct thermal behaviors associated with endothermic and exothermic transitions as a function of temperature, reflecting the effect of peroxide addition on the energetic characteristics of the formulations (Figure 5b). At lower temperatures (at 60 °C and 135 °C), all three samples exhibit small endothermic transitions, which can be attributed to the removal of physically adsorbed water and the phase transitions of ammonium nitrate (V)-(AN). Pure AN is known to undergo polymorphic transformations in this temperature range, particularly the IV → III transition near 125 °C and the III → II transition around 165 °C [32,33,38,47,53,54,55,56]. These features are visible across all samples, although the intensity of the peaks varies slightly, suggesting that hydrogen peroxide content may influence the extent of crystalline rearrangement. Between 200 and 250 °C, stronger exothermic peaks are observed, with ANFO showing the most pronounced deviation. This region is typically associated with the onset of the decomposition of AN in the presence of organic fuel (fuel oil in this case) [33,38,47,53,54,55,56]. The presence of hydrogen peroxide, which acts as an oxidizing agent, appears to accelerate the decomposition, thereby shifting the exothermic peak and intensifying its magnitude [57]. ANFO (Figure 5b) after AN melting, a pronounced, sharp exotherm occurs with a peak near approximately 295–305 °C, consistent with the literature values for AN decomposition and ANFO combustion. The relatively late onset and high peak height (largest negative excursion among the three) indicate a rapid, runaway redox once intimate contact between molten AN and FO is achieved. Adding a modest amount of H_2_O_2_ causes (ANFO-HP-5, Figure 5b) a slightly earlier exothermic onset, a broader main peak, and a reduced peak magnitude compared with ANFO. Mechanistically, H_2_O_2_ contributes additional oxidizing equivalents and active-oxygen radicals that promote AN decomposition/FO oxidation at lower temperatures, but the associated water also dilutes the oxidizer melt and absorbs heat, which tends to broaden and somewhat flatten the peak [58,59,60]. Further addition of H_2_O_2_ results in the onset shifting further to a lower temperature, and the exotherm becomes still broader. The very earliest shoulder beginning just above AN melting suggests initiation by peroxide-assisted AN melt reactions. The peak height remains below ANFO despite substantial reaction because aqueous H_2_O_2_ introduces additional heat capacity and evaporation loss, part of the heat release is distributed into the earlier temperature range, and the self-decomposition of H_2_O_2_ can proceed in parallel, partially decoupled from the fastest AN/FO pathway. Overall, ANFO-HP-10 shows the greatest “thermal sensitivity” (lowest onset) but the least violent single-peak signature [6,7,8]. Furthermore, in the range of 350–700 °C (weak baseline drift). Only low-level exothermic drift is visible, consistent with slow secondary reactions/oxidation of residual carbonaceous material once the principal decomposition is complete [16,60]. This suggests that the major decomposition processes are complete by approximately 350 °C, with only residual carbonaceous oxidation and slow degradation occurring thereafter. The convergence of the baseline indicates that hydrogen peroxide mainly affects the primary decomposition stages, but has a negligible influence at higher temperatures. Furthermore, in contrast to ANFO, the addition of HP resulted in an additional endothermic peak at ~80 °C (Figure 5b). The peak at 80 °C is likely attributable to the evaporation of surface-adsorbed water and hydrogen peroxide. The exothermic transitions are presumed to result from the decomposition of hydrogen peroxide [61].

The DSC results have indicated that HP acts as a reactive oxidizer and process modifier. It supplies active oxygen, which lowers the effective onset, while its water content and its own decomposition kinetics broaden/attenuate the peak. In complex AN-based mixtures, peroxide-related additives often shift DSC exotherms to lower T and modify apparent heat [16,58,59,60].

Importantly, the incorporation of aqueous hydrogen peroxide, substituting a portion of the fuel oil, resulted in a decreased intensity of the endothermic peak associated with ANFO decomposition. This reduction was particularly notable in ANFO-HP-10, which contained a higher concentration of hydrogen peroxide.

The theoretical calculations of the non-ideal high-energy material properties were made according to the modified BKW model and are presented in Table 2.

The theoretical calculations of the blasting properties presented in Table 2 were performed under the assumption of a constant density of 0.82 kg·dm^−3^. This simplification was adopted due to the insignificant density variations observed with the gradual substitution of ammonium nitrate (V) and fuel by hydrogen peroxide. As demonstrated by the DSC analysis, the thermal decomposition of ANFOHP formulations is highly exothermic. Moreover, the progressive replacement of AN with HP leads to an increased emission of water vapor, which can influence both the total reaction energy and the volume of gaseous products.

The enthalpy of formation of energetic materials (Table 2) represents the net energy change associated with the synthesis of the compound from its constituent elements. A higher (less negative or more positive) enthalpy of formation generally indicates a material capable of releasing greater amounts of energy upon detonation. Progressive substitution of AN with 60% HP at levels of 5% and 9.5% results in an increase in the specific enthalpy of formation from −4162.35 kJ·kg^−1^ (ANFO) to −4415.58 kJ·kg^−1^ (ANFO-HP-5) and −4694.17 kJ·kg^−1^ (ANFO-HP-10), as presented in Table 2. This effect arises because hydrogen peroxide, being a stronger oxidizing agent and possessing a higher energy content per unit mass than ammonium nitrate (V), undergoes highly exothermic decomposition into oxygen and water [22,62]. Consequently, increased incorporation of H_2_O_2_ enhances chemical energy release during detonation.

According to Chapman–Jouguet (CJ) detonation theory, the VOD is directly dependent on the heat of the reaction. Thus, a higher enthalpy of formation is expected to accelerate the detonation front as the shock wave propagates faster through the energetic medium [29,63,64,65]. The CJ framework further establishes that detonation proceeds at a unique velocity such that detonation products attain sonic flow conditions at the CJ point. This interrelates the enthalpy of formation, detonation velocity, pressure, and temperature, while simultaneously defining the Hugoniot locus for the system [64]. Consequently, a higher enthalpy of formation is associated with greater detonation pressures, since pressure correlates with the energy density of the detonation products. Enhanced energy release drives stronger expansion behind the shock front, thereby raising detonation pressure and improving explosive brisance and fragmentation potential. Similarly, detonation temperature is directly governed by the conversion of chemical energy into heat. An increased enthalpy of formation, therefore, results in elevated detonation temperatures, promoting faster and more complete reaction kinetics. It should be noted, however, that due to the negligible variation in bulk density, the overall density of the explosive mixture was assumed to be constant in the subsequent analysis of temperature, pressure, and fume volume.

Furthermore, theoretical modeling, as shown in Table 2, indicates that the gradual substitution of AN by HP results in an initial plateau followed by a slight decrease in detonation temperature, declining from approximately 2620 °C (ANFO) to 2593 °C (ANFO-HP-5) and further to 2551 °C (ANFO-HP-10). This trend may be attributed to the thermodynamic characteristics of H_2_O_2_, which, while releasing more heat per mole of oxygen, also generates greater quantities of water vapor upon decomposition. This vapor absorbs part of the released thermal energy as latent heat, thereby moderating the temperature increase. However, it is anticipated that when HP constitutes less than 5% of the composition, a slight increase in detonation temperature may occur during the initial phase of substitution. A similar trend was observed in detonation pressure. The introduction of HP into the ANFO initially causes a marginal increase in detonation pressure from 5.15 GPa (ANFO) to 5.16 GPa (ANFO-HP-5). Further substitution leads to a slight decrease, reaching 5.10 GPa for ANFO-HP-10 (Table 2). This behavior aligns with the principles of the Chapman–Jouguet (CJ) theory, highlighting the interdependence between detonation pressure, the quantity of gaseous products, and the overall reaction enthalpy. At lower substitution levels, the additional oxygen available from HP enhances the reaction energy. However, at higher HP concentrations, the energy contribution may plateau or diminish due to dilution effects, such as excess water vapor or incomplete combustion. Furthermore, the velocity of detonation (VOD) demonstrates a slight downward trend, decreasing from approximately 4815 m·s^−1^ (ANFO) to 4798 m·s^−1^ (ANFO-HP-10), as shown in Table 2. This observation reflects the sensitivity of VOD to both the energy output and the reaction kinetics. Although hydrogen peroxide decomposes more readily than ammonium nitrate (V), practical formulation challenges, sensitivity issues, and potential side reactions may limit gains in VOD, particularly in non-ideal energetic materials where density remains constant. A minor increase in the volume of gaseous products was also observed. Gas volume rose slightly from 1057 dm^3^ (ANFO) to 1059 dm^3^ (ANFO-HP-5) upon initial HP addition and then to 1064 dm^3^ (ANFO-HP-10). However, this increase was insignificant, and overall, the gas output stabilized at approximately 1060 dm^3^ per kilogram of high-energy material. Despite the stable gas yield, it is expected that the gradual substitution of the concentrated HP for AN significantly alters the fume composition. Traditional ANFO primarily produces NO_x_ and CO_x_, whereas HP decomposition results in water and oxygen, substantially increasing the water vapor fraction in detonation fumes. This shift is highly dependent on the fuel-to-oxidizer ratio, known as oxygen balance, and the detonation efficiency. Additionally, HP-based mixtures may present a more compact and efficient reaction zone, potentially aligning actual performance more closely with CJ predictions. Nevertheless, the sensitivity and thermal stability of such formulations must be carefully controlled to avoid premature decomposition or suboptimal fuel–oxidizer interaction. In formulations with a positive oxygen balance, elevated NO_x_ emissions may occur due to surplus oxygen availability.

Compression energy is defined as the energy required to compress detonation products from their initial density to the CJ state. The oxygen-rich environment created by partial substitution with H_2_O_2_ promotes more complete and exothermic combustion of the fuel fraction, yielding higher-pressure detonation products that demand greater compression energy to achieve sonic flow at the CJ plane. Experimental calculations indicate that substitution of 5% H_2_O_2_ increases compression energy slightly, from 853.83 kJ·kg^−1^ (ANFO) to 856.93 kJ·kg^−1^ (ANFO-HP-5), as shown in Table 2. However, further substitution to 10% H_2_O_2_ decreases the value to 841.21 kJ·kg^−1^ (ANFO-HP-10). These results suggest that moderate incorporation of hydrogen peroxide enhances compression energy, while excessive substitution may induce diminishing returns or secondary effects that lower compressibility.

Overall, theoretical calculations support the conclusion that gradual substitution of ammonium nitrate (V) with 60% hydrogen peroxide enhances the enthalpy of formation and, correspondingly, the energy release upon detonation. Within the Chapman–Jouguet framework, this effect manifests as elevated detonation pressure, velocity, and temperature, up to an optimal substitution level. The resulting increase in compression energy strengthens the shock wave, thereby improving detonation performance while preserving the sonic choke condition intrinsic to steady detonation waves [66,67,68,69]. It is essential to consider the non-ideal nature of these formulations and the potential discrepancies between theoretical predictions and in situ measurements [70,71]. For instance, Table 2 indicates a calculated VOD of approximately 4800 m·s^−1^, whereas the literature reports typical VOD values for conventional ANFO ranging from 1800 to 3300 m·s^−1^ in bulk form, or up to 3500 m·s^−1^ when high-energy material is in a booster [72,73,74,75]. Conversely, hydrogen peroxide-based energetic materials are reported to achieve VOD values in the range of 4500–5000 m·s^−1^ [76]. These deviations, driven by the complex and non-ideal behavior of such compositions, emphasize the need for caution when interpreting theoretical predictions and underscore the importance of future experimental validation under realistic conditions.

## 4. Conclusions

The results of this study demonstrate that hydrogen peroxide can be preliminarily applied to high-energy materials without adversely affecting the morphology of ammonium nitrate (V). SEM analysis confirmed that the FOHP blend forms a uniform thin-film coating over the AN surface, effectively masking surface deformations without causing any degradation of the AN crystalline structure. Furthermore, no evidence of droplet formation (dribbling) or phase separation (delamination) of the FOHP blend was observed. These findings were corroborated by XRD analysis, which revealed that the FOHP mixture does not chemically or structurally interact with the AN crystals, thereby preserving their original crystallinity. TG/DSC analysis indicated the additional presence of an endothermic peak at approximately 80 °C in the ANFOHP samples. This peak is likely associated with the evaporation of surface-adsorbed water and hydrogen peroxide. Moreover, partial substitution of fuel oil with aqueous hydrogen peroxide resulted in a reduction in the intensity of the endothermic peak related to ANFO decomposition, suggesting modified thermal behavior due to the presence of HP.

IR analysis confirmed that the FOHP additive did not result in the formation of new bands. The validation result after 24 h of storing indicated that all samples had records similar to IR records before storing.

Theoretical calculations further suggest that gradual substitution of hydrogen peroxide for ammonium nitrate (V) may enhance certain detonation parameters. Within the Chapman–Jouguet framework, this manifests as higher detonation pressure, velocity, and temperature, with improvements sustained up to an optimal level of substitution. The associated rise in compression energy reinforces the strength of the shock wave, thereby enhancing overall detonation performance while maintaining the sonic choke condition characteristic of steady-state detonation. However, these improvements reach a plateau or may even diminish at higher substitution levels, likely due to the increased water vapor content and potential instability of the formulation. It is therefore essential to conduct in situ measurements to validate the theoretical predictions under real-world conditions.

Of particular significance is the anticipated alteration of the post-detonation fume profile upon the inclusion of the FOHP blend. Specifically, a portion of NO_x_ is expected to be replaced by water vapor resulting from HP decomposition. This shift could lead to a substantial reduction in the toxicity of detonation fumes, offering noteworthy environmental and occupational health advantages. Nevertheless, it must be emphasized that concentrated hydrogen peroxide can pose significant risks to the human body. Therefore, the development and implementation of stringent safety protocols for its handling and use are imperative.

## Figures and Tables

**Table 1:** Chemical composition of the tested non-ideal high-energy material samples. AN—ammonium nitrate, FO—fuel oil, HP—hydrogen peroxide.

Sample Name	Component, wt.%		
	AN	FO	HP
ANFO	94	6	0
ANFO-HP-5	89	6	5
ANFO-HP-10	83.5	6.5	10.0

**Table 2:** Calculated properties of non-ideal high-energy materials.

Parameter	ANFO	ANFO-HP-5	ANFO-HP-10
Energy of formation, kJ·kg^−1^	−4162.35	−4415.58	−4694.17
Detonation temperature, °C	2620.45	2593.40	2551.843
Detonation pressure, GPa	5.15	5.16	5.10
Compression energy, kJ·kg^−1^	853.83	856.93	841.21
Velocity of detonation, m·s^−1^	4814.58	4807.68	4798.26
Volume of gas products, dm^3^	1057	1059	1064
Oxygen balance, %	0	−0.58	−0.12

## Data Availability

The original contributions presented in this study are included in the article. Further inquiries can be directed to the corresponding authors.
